# Disruption of *asxl1* results in myeloproliferative neoplasms in zebrafish

**DOI:** 10.1242/dmm.035790

**Published:** 2019-05-07

**Authors:** Evisa Gjini, Chang-Bin Jing, Ashley T. Nguyen, Deepak Reyon, Emma Gans, Michiel Kesarsing, Joshua Peterson, Olga Pozdnyakova, Scott J. Rodig, Marc R. Mansour, Keith Joung, A. Thomas Look

**Affiliations:** 1 Department of Pediatric Oncology, Dana-Farber Cancer Institute, Harvard Medical School, Massachusetts 02215, USA; 2 Molecular Pathology Unit, Center for Computational and Integrative Biology, and Center for Cancer Research, Massachusetts General Hospital, Charlestown, Massachusetts 02129, USA; 3 Department of Pathology, Harvard Medical School, Boston, Massachusetts 02115, USA; 4 Department of Pathology, Brigham and Women's Hospital, Boston, Massachusetts 02115, USA; 5 Department of Haematology, UCL Cancer Institute, University College London, London WC1E 6AG, United Kingdom

**Keywords:** Apoptosis, Hematopoietic stem cells, Myeloproliferative neoplasms, Tet2, Genome editing

## Abstract

Somatic loss-of-function mutations of the additional sex combs-like transcriptional regulator 1 (*ASXL1*) gene are common genetic abnormalities in human myeloid malignancies and induce clonal expansion of mutated hematopoietic stem cells (HSCs). To understand how *ASXL1* disruption leads to myeloid cell transformation, we generated *asxl1* haploinsufficient and null zebrafish lines using genome-editing technology. Here, we show that homozygous loss of *asxl1* leads to apoptosis of newly formed HSCs. Apoptosis occurred via the mitochondrial apoptotic pathway mediated by upregulation of *bim* and *bid*. Half of the *asxl1^+/^^−^* zebrafish had myeloproliferative neoplasms (MPNs) by 5 months of age. Heterozygous loss of *asxl1* combined with heterozygous loss of *tet2* led to a more penetrant MPN phenotype, while heterozygous loss of *asxl1* combined with complete loss of *tet2* led to acute myeloid leukemia (AML). These findings support the use of *asxl1^+/^^−^* zebrafish as a strategy to identify small-molecule drugs to suppress the growth of *asxl1* mutant but not wild-type HSCs in individuals with somatically acquired inactivating mutations of *ASXL1*.

## Abstract (precis)

**Summary:** Homozygous loss of *asxl1* in zebrafish leads to apoptosis of newly formed HSCs by upregulation of *bim* and *bid*. Half of the *asxl1^+/−^* zebrafish had MPNs by 5 months of age.

## INTRODUCTION

*ASXL1* is one of three mammalian homologs of the *Drosophila Asx* gene, which is highly conserved across multiple species (Fisher et al., 2006). Its product is an epigenetic scaffolding protein that binds to chromatin and recruits polycomb repressive complex 2 (PRC2; consisting of EZH2, EED and SUZ12), which regulates the expression pattern of developmental genes in both hematopoietic and nonhematopoietic systems (Scheuermann et al., 2010). The PRC2 complex catalyzes locus-specific trimethylation of lysine 27 on histone H3 (H3K27me3), a hallmark repressive modification that recruits the PRC1 complex (Abdel-Wahab et al., 2012a), placing further repressive modifications on chromatin by catalyzing the monoubiquitination of lysine 119 on histone H2A (H2AK119). The transcriptional repression of polycomb group (PcG) target genes by PRC1 and PRC2 is important for the maintenance of lineage-specific gene expression programs (Müller and Verrijzer, 2009; Pietersen and van Lohuizen, 2008; Schuettengruber et al., 2007; Schwartz and Pirrotta, 2007). ASXL1 also associates with the deubiquitinating enzyme BRCA1-associated protein 1 (BAP1), which removes the ubiquitin moiety from H2AK119, thereby promoting the expression of key target genes (Abdel-Wahab et al., 2012a; Scheuermann et al., 2010).

*ASXL1* is mutationally altered in several malignant myeloid diseases, including myeloproliferative neoplasms (MPNs; ∼10-15%), myelodysplastic syndromes (MDS; ∼15-25%), chronic myelomonocytic leukemia (CMML; ∼45%), and *de novo* (6.5%) or secondary (30%) cases of acute myeloid leukemia (AML) (Abdel-Wahab et al., 2011; Bejar et al., 2011; Boultwood et al., 2010a,b; Gelsi-Boyer et al., 2012, 2010; Inoue et al., 2013). These genetic alterations comprise either focal deletions, nonsense mutations or insertions/deletions (indels) that lead to frameshifts. Mutations in this gene are consistently associated with adverse outcomes, and thus serve as independent prognostic markers (Bejar et al., 2011; Gelsi-Boyer et al., 2012, 2010). In preclinical murine models, the combined loss of *Asxl1* and *Tet2* from specific hematopoietic cells resulted in an accelerated onset of MDS (Abdel-Wahab et al., 2013).

Recent reports have called attention to the fact that nonsense and frameshift mutations of ASXL1 in human myeloid malignancies often truncate the protein after 404 to 800 amino acids, retaining the ASX homology domain and truncating off the remainder of this 1541 amino acid protein, including the plant homeodomain (PHD) domain (Asada et al., 2018; Balasubramani et al., 2015; Hsu et al., 2017; Inoue et al., 2016; Kitamura, 2018; Metscher and Ahlberg, 1999; Nagase et al., 2018; Yang et al., 2018). A transgenic mouse model that expresses a truncated Asxl1 protein exhibited a gain-of-function alteration and induced myeloid malignancies (Yang et al., 2018). However, a knock-in mouse expressing a truncated ASXL1 mutant did not exhibit myeloid cell deficiency or malignancy (Hsu et al., 2017), raising the question of overexpression artifacts in the transgenic mouse model. Complicating the interpretation has been a problem in raising antibodies that recognize amino-terminal epitopes of ASXL1, so that western blotting has been very difficult and detailed biochemistry impossible, even in cell lines with classic exon 11 or 12 mutations. Heterozygous *ASXL1* mutations that occur in earlier exons have been documented in primary chronic myelomonocytic leukemia samples (Abdel-Wahab et al., 2011), suggesting that this subset have true loss of one allele, and thus that haploinsufficiency also can contribute to the onset of hematologic malignancy. Thus, further work needs to be done to separate the roles of haploinsufficiency from possible dominant-negative or gain-of-function activities that may result from different classes of ASXL1 mutations.

In humans, *de novo* constitutive heterozygous nonsense mutations in *ASXL1* have been identified in patients with Bohring-Opitz syndrome, a pediatric disease associated with developmental defects. In murine models, the hematopoietic phenotypes of animals with loss of *Asxl1* are variable. Fisher et al. reported that haploinsufficiency of *Asxl1* caused mildly perturbed myelopoiesis, while complete knockout induced severe hematopoietic defects and perinatal lethality but did not trigger the development of hematologic malignancies (Fisher et al., 2010). In another study, animals with hematopoietic-cell-specific homozygous loss of *Asxl1* developed progressive myelodysplasia culminating in MDS, an outcome that was attributed to loss of PRC2-mediated trimethylation of H3K27 (Abdel-Wahab et al., 2012a, 2013). Moreover, *Asxl1* loss cooperated with activated NRAS in this model to stimulate the rapid development of AML (Abdel-Wahab et al., 2012a). Finally, Wang et al. reported that surviving mice with ubiquitous loss of *Asxl1* had features of MDS, while *Asxl1* heterozygotes developed a milder form of the disease (Wang et al., 2014).

Here, we established zebrafish lines with germline inactivating mutations of *asxl1* early in the coding sequence of the gene and analyzed the effect of *asxl1* deficiency on hematopoietic cells in both embryonic and adult zebrafish. We show that *asxl1* is important for the survival of newly formed hematopoietic stem and progenitor cells (HSPCs) as they migrate into the caudal hematopoietic tissue of the zebrafish embryo, and that homozygous loss of *asxl1* induces mitochondrial apoptosis of HSPCs through the activation of *bim* and *bid*. In fish with heterozygous *asxl1* inactivation, half of the 5-month-old adults exhibited MPN. Among fish that were compound heterozygous for *asxl1* and *tet2*, 80% developed MPN, indicating that haploinsufficiency for *tet2* potentiates the transforming activity of *asxl1* during the pathogenesis of MPN.

## RESULTS

### Generation of *asxl1* mutant zebrafish lines by genome editing

**Fig. 1.**
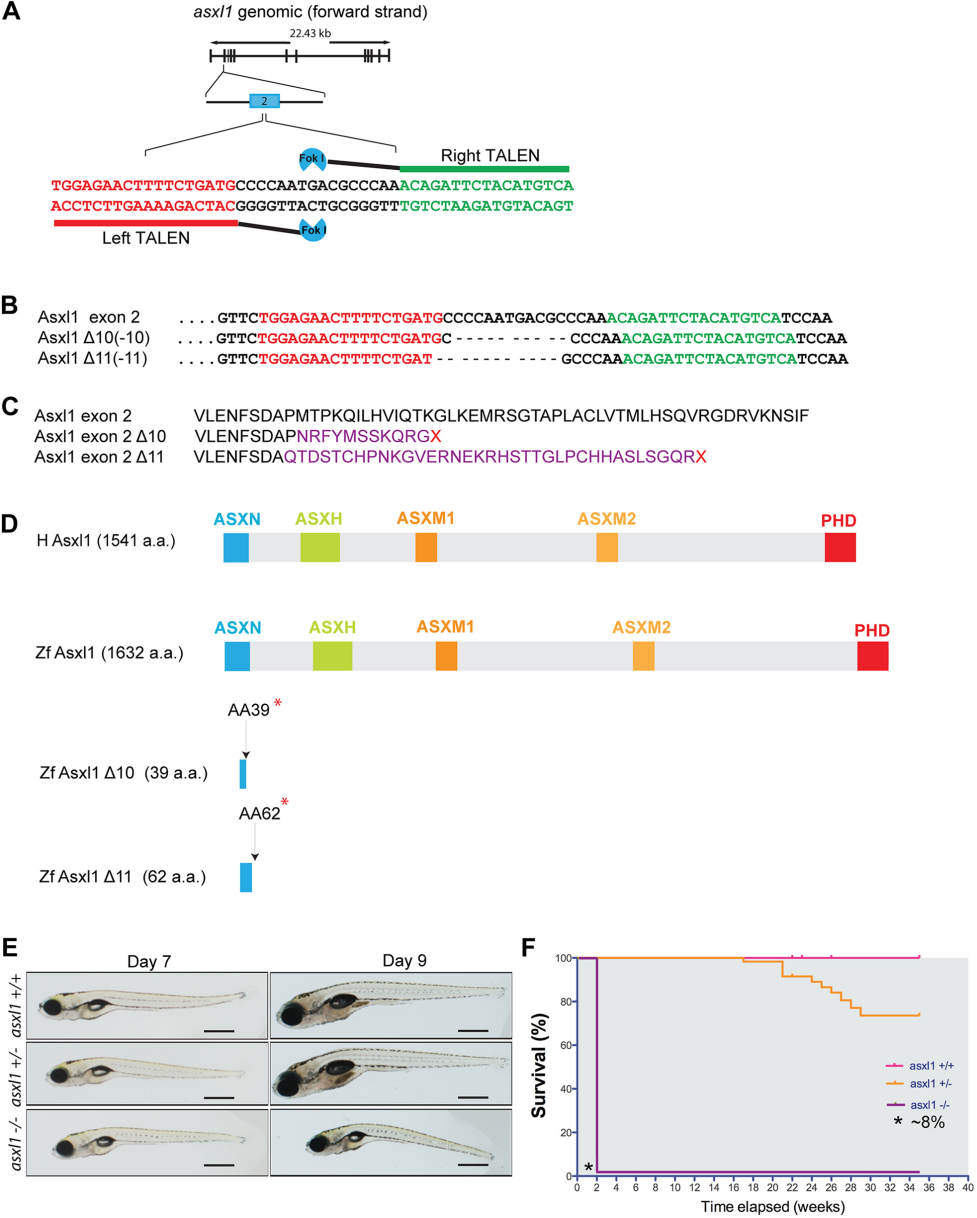
**Genome editing with use of transcription activator-like effector nucleases (TALENs) to generate null alleles of the zebrafish *asxl1* gene.** (A) Site-specific targeting for TALEN-directed Fok1 cleavage within exon 2. (B) Nucleotide sequence alignment of the *asxl1Δ10* and *asxl1Δ11* zebrafish lines compared to wild-type *asxl1*. Left, TALEN binding site appears in red; right, TALEN binding site appears in green. Dashes in the DNA sequence represent the nucleotides that are deleted during repair of Fok1-induced DNA double-stranded breaks. (C) Targeted Fok1-induced mutagenic lesions in *asxl1* produce frameshift mutations that lead to truncated protein products following short regions of novel amino acids, which are indicated in purple. Red ‘X’ denotes stop codons. (D) The truncated Asxl1 protein products predicted to be expressed in the *asxl1Δ10* and *asxl1Δ11* mutant lines lack all highly conserved functional domains. Red asterisks denote stop codons. (E) *asxl1^−/−^* zebrafish larvae mutants appear shorter in length and slimmer than *asxl1^+/+^* and *asxl1^+/^^−^* zebrafish larvae at day 7 and day 9 post-fertilization. Scale bars: 200 µm. (F) Kaplan–Meier survival curves for *asxl1^+/+^*, *asxl1^+/^^−^* and *asxl1^−/−^* mutants during the first 35 weeks of life. Asterisk (*) at the base of first curve indicates that ∼8% of *asxl1^−/−^* fish grow to normal size and survive.

The zebrafish genome contains a single zebrafish ortholog of human *ASXL1*. Alignment of the predicted zebrafish and human ASXL protein sequences showed a high level of conservation within the important functional domains (ASXN, ASXH, ASXM1, ASXM2 and PHD) that are crucial for the ability of the protein to promote PRC2-mediated repressive chromatin alterations through H3K27 trimethylation (Abdel-Wahab et al., 2012a; Fisher et al., 2006; Scheuermann et al., 2010) (Fig. S1). To generate loss-of-function alleles of the zebrafish *asxl1* gene, we designed transcription activator-like effector nucleases (TALENs) to induce premature stop codons within the sequences encoding the catalytic ASXN domain (Fig. 1A). One-cell-stage embryos were microinjected with the TALEN mRNAs, grown to maturity and subsequently outcrossed. F1 progeny were then analyzed to identify fish harboring inherited indels that disrupted the *asxl1* reading frame. We then generated two independent mutant alleles of *asxl1* (*asxl1Δ10* and *asxl1Δ11*) that possess 10- and 11-base-pair deletions, respectively, within exon 2 (Fig. 1B). Both TALEN-induced deletions led to premature stop codons (Fig. 1C) that disrupted the ASXN domain and abolished the ASXH, ASXM1, ASXM2 and PHD domains (Fig. 1D). Thus, the *asxl1Δ10* and *asxl1Δ11* lines are functionally null for *asxl1*. When all planned experiments were repeated with each mutant line, similar results were obtained. For simplicity, we present only data derived from the *asxl1Δ10* line, hereafter referred to as *asxl1^−/−^.*

### Asxl1 supports the development and viability of zebrafish

**Fig. 2.**
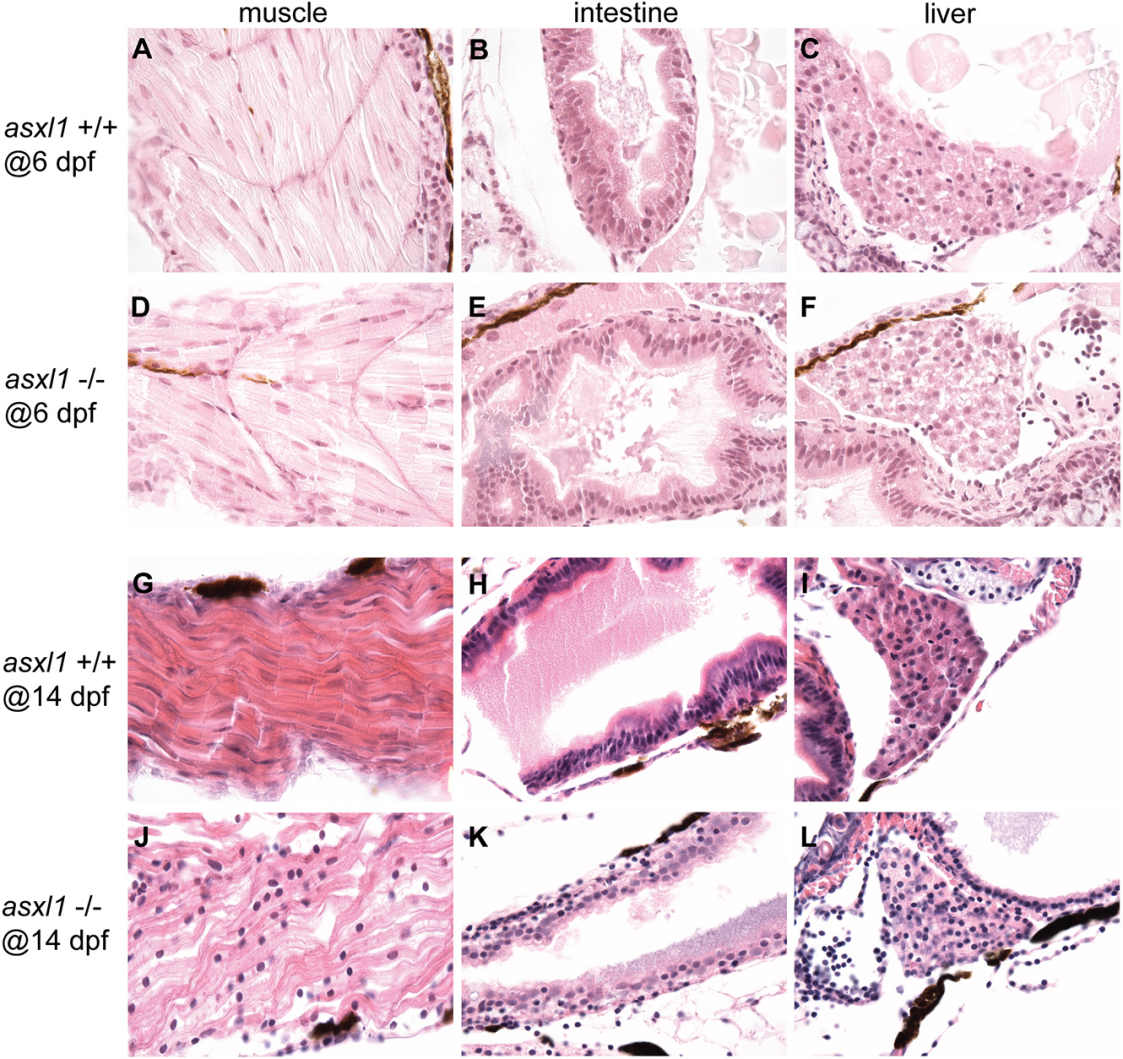
***Asxl1* loss affects organ development.** Histopathological analysis of the muscle, intestine and liver development with hematoxylin and eosin staining in 5 *asxl1^+/+^* and 5 *asxl1^−/−^* mutants at 6 dpf and 14 dpf. Muscle (A,D) and intestine (B,E) development had no difference between *asxl1^+/+^* and *asxl1^−/−^* embryos at 6 dpf. Liver parenchyma at 6 dpf appeared abnormal with vacuolated cells in *asxl1^−/−^* embryos (F) compared with *asxl1^+/+^* embryos (C). At 14 dpf, muscular atrophy was shown in *asxl1^−/−^* embryos (J) compared with normal striated muscle in *asxl1^+/+^* embryos (G). Intestine in *asxl1^−/−^* embryos was abnormal with villus blunting (K) compared with normal nuclei and microvilli in the *asxl1^+/+^* embryos (H). The *asxl1^−/−^* embryos exhibited progressive liver architectural distortion (L) compared to *asxl1^+/+^* embryos (I).

To determine whether *asxl1* is important for the growth and viability of developing zebrafish, we examined the morphology of clutches of fish derived from crosses of two heterozygous zebrafish. We found that *asxl1*^+/−^ fish develop normally and are indistinguishable from *aslxl1*^+/+^ fish during development, although they died prematurely starting at about 18 weeks of age (Fig. 1F). By contrast, *asxl1*^−/−^ fish were viable but appeared abnormal by 7 days post-fertilization (dpf), when 100% of mutants (27 of 27) were shorter in length and had a reduced dorsal-ventral size overall. These homozygous mutant fish did not grow by 9 dpf (Fig. 1E), and the vast majority (25 out of 27) had died by 14 dpf (Fig. 1F). To explore the death mechanism of *asxl1*^−/−^ embryos, we studied the organ development at 6 dpf and 14 dpf by histopathologic analysis. At 6 dpf, the *asxl1*^−/−^ embryos exhibited normal morphology for the muscle and intestine as compared with *asxl1^+/+^* embryos (Fig. 2A,D and B,E). However, by 6 dpf the liver parenchyma appeared abnormal in *asxl1*^−/−^ embryos, with atypical hepatocytes containing poorly demarcated cells with vacuolated cytoplasm (Fig. 2C,F). By 14 dpf, the *asxl1*^−/−^ zebrafish embryos showed muscular atrophy with disorganization of muscle fibers and loss of differentiation as evidenced by round and not elongated nuclei compared with the normal striated muscle fibers and elongated nuclei in the muscle of *asxl1*^+/+^ fish (Fig. 2G,J). The intestine was also abnormal in *asxl1*^−/−^ embryos, which showed intestinal architectural atypia with significant villus blunting and disorganized localization of cell nuclei, when compared to the normal cell nuclei and microvilli in the *asxl1*^+/+^ intestinal epithelium (Fig. 2H,K). Additionally, the 14 dpf *asxl1*^−/−^ embryos examined demonstrated progressive architectural distortion of the liver parenchyma as evident by widespread atypical hepatocytes with cell crowding and poor cell border demarcation when compared to the *asxl1^+/+^* embryos (Fig. 2I,L).

It seems plausible that the relative hypoplasia of the intestinal epithelium might account in part for the runted overall appearance of the juvenile *asxl1*^−/−^ fish, because the intestinal villus normally increases the mucosal surface 10-fold and the microvilli that make up the normal brush border increase the absorptive surface by 20-fold. Of course, we cannot establish the precise cause of the failure to thrive and premature death of the majority of *asxl1*^−/−^ embryos based on histology alone. However, we suspect that it can be reversible, because the small percentage of *asxl1*^−/−^ fish that survive are initially as small as the other *asxl1*^−/−^ fish, but, if they survive, they gradually attain normal size by 5 months of age and both males and females are fertile. Histopathological analysis of 17-month-old *asxl1*^−/−^ mutants revealed that the intestine and liver were normal (Fig. S2D-I), indicating that these fish had recovered from early developmental hypoplasia in these organs (Fig. 2). However, they did have decreased numbers of erythroid islands in the kidney marrow (Fig. S2A-C), indicating that the hematopoietic system was abnormal. Thus, *asxl1* is almost always essential for development and viability, although rare individuals can recover normal size and survive well into adulthood.

### Asxl1 is required for definitive hematopoietic stem and progenitor cell survival

**Fig. 3.**
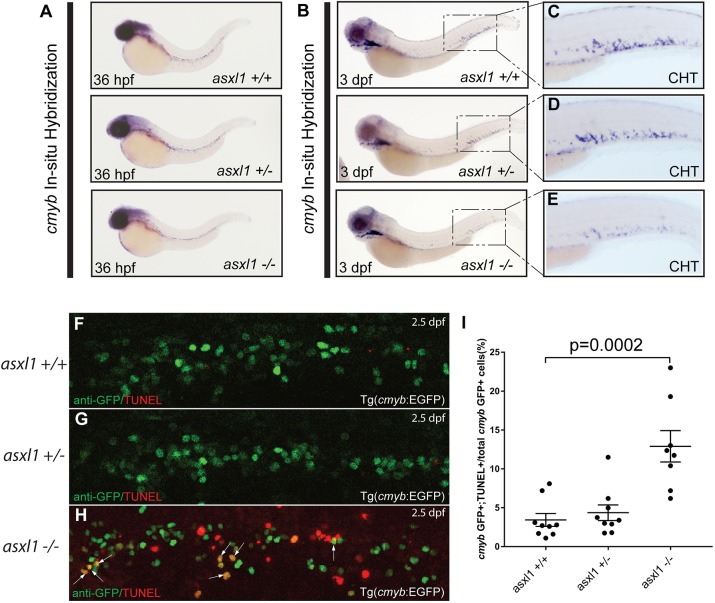
**Loss of *asxl1* induces apoptosis in *cmyb*-expressing embryonic HSPCs.** WISH for *cmyb* was performed at 36 hpf (A) and 3 dpf (B-E) in *asxl1^+/+^*, *asxl1^+/^^−^* and *asxl1^−/−^* zebrafish embryos*.* Boxes in panel B are shown at higher magnification in C-E. (F-H) HSPCs (GFP; green) and cells undergoing apoptosis (TUNEL, TMR red, Roche) in the CHT of 48-hpf *asxl1^+/+^*, *asxl1^+/^^−^* and *asxl1^−/−^* zebrafish embryos in the *Tg(cmyb:EGFP)* reporter line background were identified by immunofluorescence microscopy following a dual TUNEL/anti-GFP assay. Arrows indicate apoptotic HSPCs with combined EGFP and DMR red fluorescence signals. (I) *cmyb*:GFP+/TUNEL+ cells in *asxl1^+/+^*, *asxl1^+/−^* and *asxl1^−/−^* were quantified as a percentage of total *cmyb*:GFP+ cells. Bars represent the mean and s.e.m. for 8-9 embryos each. Unpaired Student’s *t*-tests and Prism software were used to determine the *P*-value for each genotypic group compared with control cells.

We next asked whether *asxl1* is required for normal embryonic and definitive hematopoiesis. In zebrafish with heterozygous or homozygous inactivation of *asxl1*, there was normal development of the embryonic erythroid, macrophage and granulocytic lineages (Fig. S3). The number of definitive HSPCs in *asxl1* mutant embryos was determined by whole-mount *in situ* hybridization (WISH) with a *cmyb* riboprobe at both 36 hpf (Fig. 3A) and 3 dpf (Fig. 3B-E) on the progeny of a cross between heterozygous *asxl1* mutant fish. At 36 hpf, the number of newly formed HSPCs budding from the ventral wall of the dorsal aorta appeared normal regardless of genotype (Fig. 3A); however, at 3 dpf there was an apparent decrease in the number of cells expressing *cmyb* in the caudal hematopoietic tissue (CHT) of the *asxl1^−/−^* embryos (Fig. 3E), compared to both *asxl1* wild-type and heterozygous embryos (Fig. 3C,D). To further confirm the *cmyb* WISH results shown in Fig. 3, we crossed *asxl1*^+/−^ fish with *Tg(cmyb:EGFP)*, in which HSPCs could be visualized by EGFP. Incrosses of these fish showed a statistically significant (*P*=0.0002) reduction of EGFP+ cells at 3 dpf based on EGFP fluorescence (Fig. S4), similar to the results we observed by WISH at 3 dpf (Fig. 3). Because *cmyb+* HSPCs were dramatically decreased in *asxl1^−/−^* embryos between 36 hpf and 3 dpf (Fig. 3E), we postulated that the *asxl1^−/−^* HSPCs may be undergoing apoptosis during this interval.

To address this hypothesis, we performed terminal transferase UTP nick end labeling (TUNEL) analysis coupled with immunohistochemistry (IHC) for *cmyb*/GFP in 48-hpf *asxl1*^+/+^, *asxl1*^+/−^ or *asxl1^−/−^* embryos generated in the background of the *Tg(cmyb:GFP)* reporter line (North et al., 2007). Compared with *asxl1^+/+^* and *asxl1^+/^^−^* embryos, those with an *asxl1^−/−^* genotype showed a significant increase in the number of *cmyb*/GFP*+*/TUNEL+ cells (Fig. 3F-I). These results indicate that, although *asxl1* is not required for HSPC specification and budding from the ventral wall of the dorsal aorta at 36 hpf, it is important for the survival of newly formed HSPCs as they migrate into the CHT of the zebrafish embryo.

**Fig. 4.**
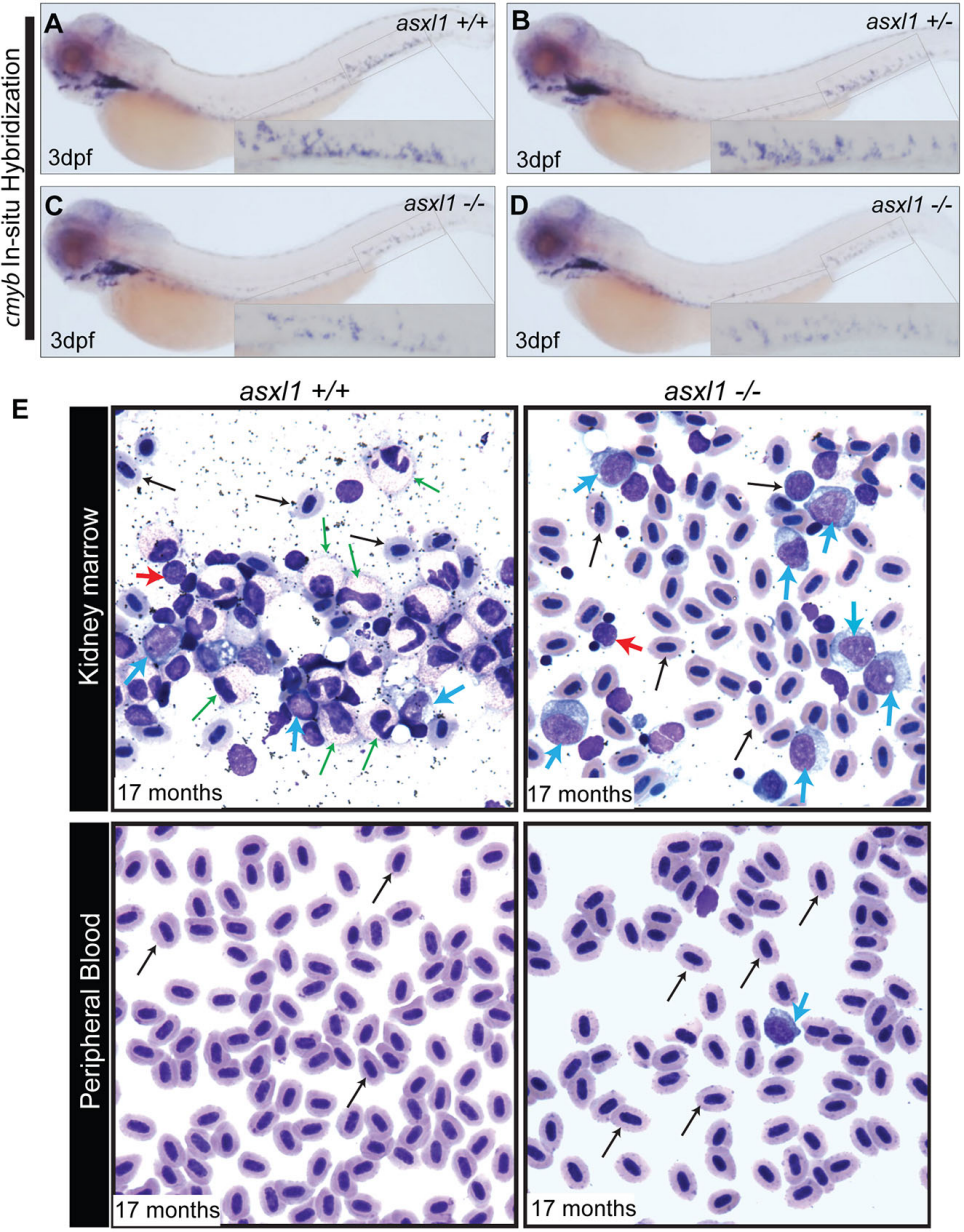
**Complete loss of *asxl1* leads to AML in zebrafish.** WISH for *cmyb* was performed at 3 dpf in *asxl1^+/+^* (A), *asxl1^+/^^−^* (B) and *asxl1^−/−^* (C) zebrafish embryos obtained from a cross of two *asxl1^+/^^−^* fish*.* (D) WISH for *cmyb* was performed at 3 dpf in *asxl1^−/−^* zebrafish embryos obtained from a cross of two *asxl1^−/−^* fish. Insets show a higher magnification of the CHT in each panel. (E) MGG staining was performed on kidney marrow smears and peripheral blood smears of *asxl1^+/+^* and *asxl1^−/−^* fish at 17 months of age. Normal maturation and morphology are shown across a spread of blood cells in the kidney marrow of the *asxl1^+/+^* zebrafish. In the *asxl1^−/−^* fish, the kidney marrow is replaced with immature myeloid blast cells in a pattern resembling AML. Circulating immature myeloid blast cells were observed in the peripheral blood of the *asxl1^−/−^* zebrafish, but not *asxl1^+/+^* fish. Erythrocytes, black arrow; myeloid cells, green arrow; blast cells, blue arrow; lymphocytes, red arrow.

To determine whether the progeny of surviving *asxl1^−/−^* adult fish had defects in hematopoiesis, we compared the progeny of incrosses of *asxl1^+/^^−^* fish with those of incrosses of *asxl1^−/−^* fish. Then, we performed WISH with a *cmyb* riboprobe at 3 dpf to assess the number of definitive HSPCs in embryos at that developmental stage. There was a similar decrease in the number of HSPCs expressing *cmyb* in the CHT of 3 dpf *asxl1^−/−^* embryos, whether they arose from heterozygous (10 of 42; Fig. 4C) or homozygous (10 of 10; Fig. 4D) incrosses, as compared to wild-type (Fig. 4A) and heterozygous *asxl1* (Fig. 4B) embryos.

We also tested whether a complete loss of *asxl1* leads to defects in hematopoiesis in adult zebrafish, as indicated by the reduction of erythroid islands in the kidney marrow, as shown for a representative *asxl1^−/−^* fish at 17 months of age (Fig. S2A-C). Giemsa staining of the hematopoietic cells from kidney marrow touch preps of seven *asxl1^−/−^* zebrafish sacrificed at 16- to 17-months of age showed a complete lack of myeloid maturation with increased numbers of myeloblasts (Fig. 4E, blue arrows) when compared to wild-type fish. In the absence of secondary causes, such as toxic exposure or drug administration, arrested myeloid maturation strongly suggests a myeloid neoplasm, and the presence of increased myeloblasts supports a diagnosis of a disease resembling AML. In peripheral blood smears from *asxl1^−/−^* fish, only rare myeloblasts were present (Fig. 4E, blue arrows), which is not an unusual feature of AML. By close examination of blood smears from two *asxl1^−/−^* fish, we identified circulating immature myeloid blast cells in the peripheral blood of the mutant zebrafish (3 of 250 blood cells in fish 1, and 4 of 250 blood cells in fish 2) that were not evident in wild-type fish (0 in 250 blood cells in fish 1, and 0 in 250 blood cells in fish 2; Fig. 4E, blue arrows). Thus, complete loss of *asxl1* leads, after 16-17 months, to a disease resembling AML in zebrafish, likely after accumulation of additional mutations or epigenetic alterations.

### Aberrant upregulated expression of *bim* and *bid* mediates the loss of HSPCs in *asxl1* mutants

**Fig. 5.**
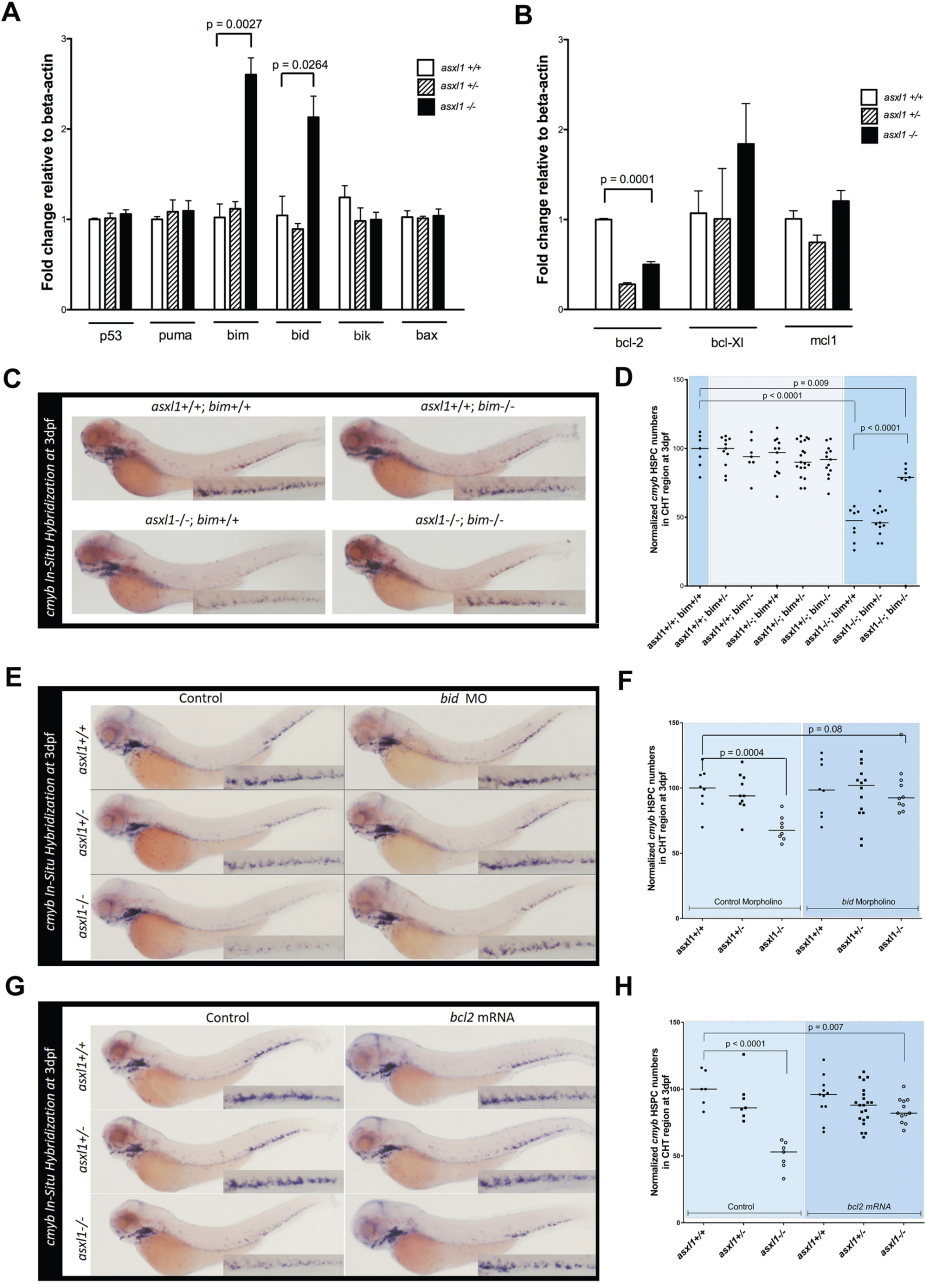
**Overexpression of *bim* and *bid* mediates apoptosis in *asxl1****^−/−^* **HSPCs.** (A,B) Quantitative PCR was performed with cDNA isolated from the trunks of 48 hpf *asxl1^+/+^*, *asxl1^+/^^−^* and *asxl1^−/−^* zebrafish embryos to quantify the expression of pro-apoptotic (A) and anti-apoptotic (B) members of the Bcl2 family. Expression levels are shown relative to β-actin. The values are means of triplicate runs with s.e.m. Statistical significance was determined with unpaired Student's *t*-test. Results from a single experiment are shown; however, four independent experiments were performed for both panels A and B with similar results. (C) WISH to detect *cmyb* with the indicated genotypes at 3 dpf, showing that the loss of HSPCs in *asxl1^−/−^* fish is rescued by loss of *bim*. (D) Cropped CHT regions from panel C were quantified with use of 98 embryos per genotype with ImageJ software. (E) WISH to detect *cmyb* was performed at 3 dpf for embryos with the indicated genotypes that were injected with 16 ng of either *bid* morpholino or control morpholino, showing that the loss of HSPCs in *asxl1^−/−^* fish is rescued by loss of *bid*. (F) Cropped CHT regions from panel E were quantified with use of 60 embryos per genotype and ImageJ software. (G) WISH to detect *cmyb* was performed at 3 dpf for embryos with the indicated genotypes that were injected with 100 ng/µl mRNA encoding either Bcl2 or GFP. Overexpression of *bcl2* rescued the loss of HSPCs in *asxl1^−/−^* fish. (H) Cropped CHT regions from panel G were quantified with use of 70 embryos per genotype and ImageJ software. In panels D, F and H, black bars representing the median values. Statistical analysis was done with Prism software. Unpaired Student's *t*-tests were performed in Prism software to determine the *P*-value for each genotype group compared to controls.

To investigate the mechanism by which HSPCs undergo apoptosis in 48 hpf *asxl1^−/−^* embryos, we performed quantitative real-time PCR (qPCR) on mRNA isolated from the CHT region of 48 hpf *asxl1^+/+^*, *asxl1^+/^^−^* and *asxl1^−/−^* zebrafish embryos (Fig. 5A,B). We analyzed expression of *p53* (*tp53*) as well as both pro-apoptotic (*puma*, *bim*, *bid*, *bik* and *bax*) and anti-apoptotic (*bcl2*, *bcl-xL* and *mcl1a*) members of the Bcl2 family. There was significant overexpression of *bim* and *bid* in *asxl1^−/−^* compared to *asxl1^+/+^* and *asxl1^+/^^−^* embryos (Fig. 5A; *P*<0.05) but no change in *p53*, *puma*, *bik* or *bax* expression. Additionally, we observed a significant decrease in *bcl2* expression but no change in the levels of *bcl-xL* or *mcl1a* in *asxl1^−/−^* embryos (Fig. 5B). These data suggest that changes in *bim*, *bid* and *bcl2* mediate HSPC apoptosis in 48 hpf *asxl1^−/−^* embryos, thereby leading to loss of *cmyb*+ cells in the CHT at 3 dpf.

To test whether *bim* is required for the loss of *asxl1^−/−^* HSPCs, we used a *bim-*mutant zebrafish line (*bcl2l11zdf19*) that was generated by retroviral insertional mutagenesis (Golling et al., 2002). This line (hereafter referred to as the *bim* mutant line) harbors a retroviral insertion located within the coding sequence of *bim* exon 2 and lies upstream of the BH3 domain required to induce apoptosis, thereby creating a loss-of-function allele of *bim*. We crossed the *bim* mutant line into our *asxl1^+/^^−^* zebrafish line and analyzed the progeny for *cmyb* expression by WISH at 3 dpf (Fig. 5C,D). Our data show that *bim* is required for the loss of *cmyb*+ HSPCs in the CHT of *asxl1^−/−^* zebrafish at 3 dpf. We next asked whether *bid* is required for apoptosis in 48 hpf *asxl1^−/−^* zebrafish HSPCs. Thus, after incrossing *asxl1^+/^^−^* adult zebrafish and injecting the progeny with a *bid*-specific splice-blocking morpholino (MO) (Kratz et al., 2006; Pyati et al., 2011), we performed WISH for *cmyb* at 3 dpf, subsequently genotyped the embryos for the *asxl1* mutation and quantified the results of the *cmyb* WISH (Fig. 5E,F). Remarkably, knockdown of *bid* also partially rescued the loss of *cmyb*+ HSPCs in the CHT of *asxl1^−/−^* zebrafish at 3 dpf.

To determine whether the decrease in *bcl2* expression could contribute to the apoptosis observed in 48 hpf *asxl1^−/−^* HSPCs, we incrossed *asxl1^+/^^−^* adult zebrafish and injected the progeny with mRNA encoding *bcl2* or a control gene. We again performed WISH for *cmyb* at 3 dpf, genotyped the embryos and quantified the results (Fig. 5G,H). We found that overexpression of *bcl2* rescued most of the apoptotic *asxl1^−/−^* HSPCs. To show these findings by confocal fluorescence microscopy, we repeated the rescue assay in *asxl1^−/−^*, *asxl1^+/−^* and *asxl1^+/+^* embryos bred into the *Tg(cmyb:EGFP)* background, in which HSPCs could be visualized by expression of EGFP. Statistically significant partial rescue of EGFP+ HSPCs was observed at 3 dpf in *asxl1^−/−^* embryos injected with each of the *bim* MO and *bid* MO, and also with *bcl2* mRNA (Fig. S5). This experiment indicated that the apoptosis that occurred shortly after budding of *asxl1^−/−^* HSPCs from the hemogenic endothelium was due to the combined action of the BH3-only proteins Bim and Bid, and that it could be blocked by overexpression of *bcl2*. Thus, death of *asxl1^−/−^* HSPCs occurred through programmed cell death mediated by the intrinsic mitochondrial apoptotic pathway.

### Combined loss of *asxl1* and *tet2* potentiates the development of MPN and leads to AML in a subset of adult zebrafish

**Fig. 6.**
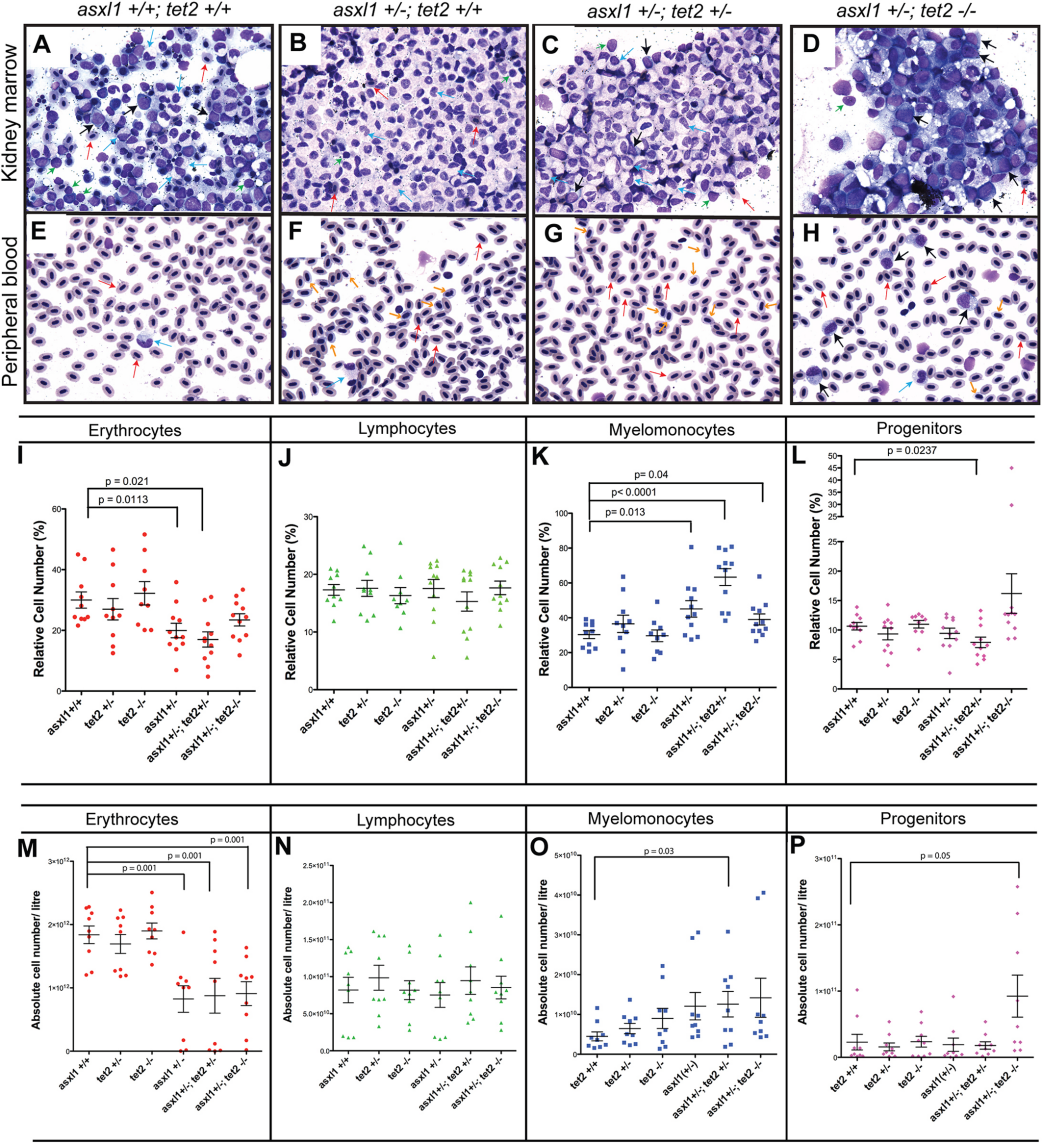
**Combined loss of *asxl1* and *tet2* leads to MPN and AML in a subset of adult zebrafish.** Morphological and quantitative analysis of blood cell types in the kidney marrow and peripheral blood of 5-month-old fish with the indicated genotypes. MGG staining was performed on kidney marrow smears. (A-H) Red arrows denote mature erythrocytes; light blue arrows denote myelomonocytes; green arrows denote lymphocytes; black arrows denote progenitor cells; and orange arrows denote immature red blood cells. (A) In wild-type fish, the kidney marrow hematopoietic cells showed normal maturation and morphology. (B) Five of 11 *asxl1^+/^^−^tet2^+/+^* fish had an increased number of mature myeloid cells, indicating MPN; the remaining 6 fish had normal morphology. (C) Eight of 11 *asxl1^+/^^−^tet2^+/^^−^* fish showed an increased number of mature myeloid cells, indicating MPN. (D) Two of 10 *asxl1^+/^^−^tet2^−/−^* fish showed increased myeloid blast cells, indicating AML. Four of the remaining 8 fish had MPN, while the other 4 had normal morphology. (E-H) Analysis of peripheral blood smears by MGG staining. (E) Wild-type fish had normal maturation and morphology of the blood cells. (F) Five of 11 *asxl1^+/^^−^tet2^+/+^* fish showed rounded circulating immature red blood cells and increased mature myeloid cells. (G) Eight of 11 *asxl1^+/^^−^tet2^+/^^−^* fish showed immature rounded circulating red blood cells. (H) Two of 10 *asxl1^+/^^−^tet2^−/−^* fish showed an increased number of myeloid blast cells, myelomonocytes and immature erythrocytes. (I-L) Forward- versus side-scatter analysis of kidney marrow cell populations in 5-month-old fish with the indicated genotypes. (M-P) Forward- versus side-scatter analysis of absolute cell numbers per liter of blood in 5-month-old fish with the indicated genotypes, showing increased myelomonocytes and decreased red blood cells in *asxl1^+/^^−^* fish. A subpopulation of *asxl1^+/^^−^tet2^−/−^* fish showed increased progenitor cell numbers in kidney marrow and blood. Mean values with s.e.m. are shown. Statistical analysis was done with Prism software. Unpaired Student's *t*-tests were performed in Prism to determine the *P*-value for each genotypic group compared with controls.

We recently described a zebrafish model of MDS in which both *tet2^−/−^* and *tet2^+/^^−^* fish develop trilineage dysplasia at 11 months of age and full-blown MDS with anemia by 24 months (Gjini et al., 2015). In human myeloid malignancies, inactivating mutations of *ASXL1* and *TET2* often occur together in the same patient's malignant cells (Abdel-Wahab et al., 2012b; Patnaik et al., 2016), suggesting that loss of these two tumor suppressors may act synergistically in myeloid transformation. To pursue this hypothesis, we intercrossed the *tet2^+/^^−^* and *asxl1^+/^^−^* lines and then inbred the compound heterozygous progeny to generate each of the genotypes that was relevant to our experiment (*asxl1^+/+^tet2^+/+^*, *asxl1^+/^^−^tet2^+/+^*, *asxl1^+/^^−^tet2^+/^^−^* and *asxl1^+/^^−^tet2^−/−^*). These progenies were identified through genotyping at 2 months of age and were closely monitored for survival. At 5 months of age, May–Grünwald–Giemsa (MGG) staining of kidney marrow and peripheral blood smears representing 9-11 individual fish per genotype (Fig. 6A-H) revealed an increase in the number of myelomonocytes (Fig. 6A-D, light-blue arrows) in the kidney marrow of a subset of animals with heterozygosity for *asxl1* [*asxl1^+/^^−^tet2^+/+^* (5 of 11); *asxl1^+/^^−^tet2^+/^^−^* (8 of 11) and *asxl1^+/^^−^tet2^−/−^* (3 of 10)], which is diagnostic of MPN. Moreover, 2 of 10 *asxl1^+/^^−^tet2^−/−^* fish had high numbers of immature myeloid cells lacking differentiation, which is indicative of AML (Fig. 6D, black arrows). Analysis of peripheral blood smears showed an aberrant increase in circulating immature red blood cells (Fig. 6E-H, orange arrows) in a subset of animals heterozygous for *asxl1* [*asxl1^+/^^−^tet2^+/+^* (5 of 11), *asxl1^+/^^−^tet2^+/^^−^* (8 of 11) and *asxl1^+/^^−^tet2^−/−^* (3 of 10)]. These results demonstrate the rapid onset of MPN in fish with heterozygous loss of *asxl1* and *tet2*, with AML apparently being restricted to *asxl1^+/^^−^* fish with loss of both *tet2* alleles.

To assess the consequences of *asxl1* heterozygosity on hematopoiesis more rigorously, we quantified 4 major categories of blood cell populations (erythrocytes, lymphocytes, myelomonocytes and progenitor cells) with respect to their relative numbers in the kidney marrow and their absolute numbers per liter of peripheral blood. Forward- and side-scatter flow cytometry was performed individually for wild type or *asxl1* heterozygous mutants, alone or in combination with hetero- or homozygosity for *tet2* (i.e. 6 genotypes, 9-11 fish per group). Analysis of the relative numbers of each cell type in the kidney marrow (Fig. 6I-L and Fig. S6) revealed a significant decrease in the erythroid cell fraction in *asxl1^+/^^−^tet2^+/+^* and *asxl1^+/^^−^tet2^+/^^−^* fish, but not in *asxl1^+/^^−^tet2^−/−^* fish, compared to wild type (Fig. 6I). The lymphocyte fraction, by contrast, did not differ appreciably, consistent with findings in Fig. 6A-D. Myelomonocytes increased significantly in *asxl1^+/^^−^tet2^+/+^*, *asxl1^+/^^−^tet2^+/^^−^* and *asxl1^+/^^−^tet2^−/−^* fish compared to wild type (Fig. 6K). Finally, there was a significant decrease in the progenitor cell fraction of *asxl1^+/^^−^tet2^+/^^−^* fish but not in those with other genotypes, compared to wild type (Fig. 6L). Analysis of absolute cell counts in peripheral blood for each blood cell type (Fig. 6M-P) supported the results based on cell fraction, with contribution of homozygous loss of *tet2* becoming more evident. Thus, regardless of *tet2* status, heterozygosity for *asxl1* consistently increased myelomonocytes in the kidney marrow and lowered absolute numbers of circulating erythrocytes, both characteristic features of human MPN. Notably, however, homozygous loss of *tet2* leads to AML in 20% of *asxl1* heterozygotes, suggesting a synergistic interaction between these two genes when they are both mutated in the same myeloid progenitors.

## DISCUSSION

The *ASXL1* gene is often somatically mutated in patients with hematopoietic malignancies such as MDS, MPN and AML, generally through acquired heterozygous nonsense or frame-shift mutations (Abdel-Wahab et al., 2011; Bejar et al., 2011; Boultwood et al., 2010a,b; Gelsi-Boyer et al., 2012, 2010; Inoue et al., 2013). Here, we report a zebrafish model with germline *asxl1* inactivation and the unique property that half of the fish with heterozygous mutations develop MPNs by 5 months of age, characterized by increased numbers of myelomonocytes in the kidney marrow and peripheral blood together with anemia. In this context, our preclinical model shows the considerable consequences *in vivo* of haploinsufficiency for a true null allele of *asxl1* in terms of myeloid malignancy, because the mutations we have made truncate the protein after amino acids 39 or 62, which is upstream of or internal to each of the conserved domains of ASXL1 (see Fig. 1D). Based on recent publications suggesting that an amino-terminal portion of ASXL1 with dominant-negative or neomorphic activity could be expressed in some patients with hematologic malignancy (Asada et al., 2018; Balasubramani et al., 2015; Hsu et al., 2017; Inoue et al., 2016; Kitamura, 2018; Nagase et al., 2018; Yang et al., 2018), it will also be important in the future to produce zebrafish models that introduce *asxl1* frame-shift mutations and premature termination in exon 11 or early in exon 12 to assess additional phenotypes that may accompany a truncated protein arising from this gene.

Abdel-Wahab and colleagues (2013) used an elegant conditional *Asxl1* knockout approach inserting two *loxP* sites that flanked exons 5-10 to generate their murine model. Hematopoietic-cell-specific homozygous deletion of *Asxl1* resulted in MDS characterized by progressive, multilineage cytopenias and dysplasia accompanied by increased numbers of less differentiated hematopoietic stem and progenitor cells. The MDS phenotype in *Asxl1* haploinsufficient mice at 6-12 months (Abdel-Wahab et al., 2013) was different from the very penetrant and early MPN phenotype (5 months) that we observed in the *asxl1* mutant zebrafish model. Thus, subtle differences exist in the myeloid phenotypes and possibly the affected downstream pathways disrupted by *asxl1* haploinsufficiency in these two vertebrate models of myeloid malignancy. The disease was transplantable, with a shorter latency for myeloid malignancies with *Asxl1* null hematopoietic cells versus those with haploinsufficiency. ASXL1 loss in this model leads to a global reduction of PRC2-mediated trimethylation of H3K27 and thus causes dysregulated expression of key regulators of hematopoiesis, including HOXA9 (Abdel-Wahab et al., 2013). In results similar to those of Abdel-Wahab et al. in mice (Abdel-Wahab et al., 2013), we show that *tet2* and *asxl1* cooperate in the pathogenesis of myeloid malignancies in zebrafish, although the phenotypes in zebrafish that we report are focused on MPN progressing to AML, rather than progressive MDS as observed in the murine model.

Wang and colleagues (2014) developed a constitutive *Asxl1* murine knockout line by disrupting the translation start site of *Asxl1*. A small fraction of the homozygous knockout mice survived for a maximum of 18-42 days, and some of these developed MDS with anemia, thrombocytopenia and neutropenia. However, heterozygous *Asxl1^+/^^−^* animals in this model developed only minimal abnormalities in myeloid cell morphology without significant alterations in blood counts, similar to the constitutive *Asxl1* knockout mice reported by Fisher et al. (2010) and Wang et al. (2014). Thus, haploinsufficiency for *ASXL1*, the main acquired genetic abnormality in human myeloid malignancies, does not by itself seem to reproducibly induce hematopoietic malignancies in mice.

Homozygous loss of *asxl1* in our zebrafish model caused profound hypoplasia of the gastrointestinal system, with underdeveloped epithelium, resulting in very small larvae and near-complete lethality by 14 dpf. Interestingly, about 8% of the *asxl1^−/−^* zebrafish larvae in our study survived, regained normal size and became fertile by 3 months of age. Thus, in the zebrafish, the total absence of *asxl1* appears to delay or prevent the emergence of pathways important for gastrointestinal development but, with time, these pathways are restored in a small fraction of the fish, enabling their unimpeded progression to normal adulthood. The hematopoietic phenotype of adult *asxl1^−/−^* fish was profound in that all of the surviving animals that we examined had kidney marrow morphology suggestive of AML by 17 months of age.

We did not observe craniofacial abnormalities or anophthalmia, both of which have been reported in the murine models with *Asxl1* deletion. For example, homozygous inactivation of *Asxl1* by Abdel-Wahab and colleagues (2013) in nearly all tissues with *EIIa-cre* caused embryonic lethality and craniofacial abnormalities, including microophthalima/anophthalmia. Similarly, Wang and colleagues (2014) reported that homozygous constitutive inactivation of *Asxl1* in mice led to dwarfism and anophthalmia. Fisher et al. (2010) also developed a constitutive loss-of-function murine model, with insertion of a *pgk* promoter-driven neomycin-resistance cassette into exon 5. In retrospect, this alteration appears to be hypomorphic, as homozygous insertion produced only modest homeotic transformation of the axial skeleton and runting.

We observed that *asxl1^−/−^* fish have defects in the earliest phases of definitive hematopoiesis, in that homozygous loss of *asxl1* led to reduced numbers of HSPCs by 72 hpf. We also noted that specification of definitive HSCs from the ventral wall of the dorsal aorta occurs normally in *asxl1^−/−^* fish, whereas the newly formed HSCs rapidly succumb to apoptosis, such that only 40% of normal numbers remain by 72 hpf. Importantly, the increased apoptosis that we observed is mediated through the mitochondrial pathway, as it can be rescued by Bcl2. We further show that the death of migratory HSPCs is induced by upregulation of the BH3-only pro-death proteins Bim and Bid, as knockout of either of these genes could partially rescue the affected HSPCs in *asxl1^−/−^* animals.

Because of the optical clarity of zebrafish embryos and juvenile fish, the *asxl1* mutant zebrafish lines reported here provide definite imaging advantages for assessing the pathways involved in the initiation and maintenance of MPNs arising from this deletion. These same advantages permit the analysis of chemical libraries of US Food and Drug Administration (FDA)-approved drugs for their ability to inhibit zebrafish HSPCs in order to discover drugs that are synthetic lethal with *asxl1* loss for the suppression of HSPC growth and survival. A very successful screen of HSPCs in zebrafish embryos by North and coworkers (2007) identified PGEII as a promoter and indomethacin as a suppressor of the growth of HSPCs in normal embryos. This same approach could be applied to identify drugs that specifically suppress HSPCs with haploinsufficiency for ASXL1. Such drugs might prove active in human MPN cases with *ASXL1*-inactivating mutations. Moreover, clonal hematopoiesis, arising in 10% of individuals over 65 years of age, has been linked to clonal expansion of HSPCs with mutations of several genes, including the epigenetic modifiers *DNMT3A*, *TET2* and *ASXL1* (Genovese et al., 2014; Jaiswal et al., 2014). Importantly, this age-related defect predisposes to the development of hematologic malignancy (Corces-Zimmerman et al., 2014; Hirsch et al., 2016; Shlush et al., 2014) and more recently was related to a doubling of the risk of coronary artery disease (Jaiswal et al., 2017). Currently, there is a lack of safe and efficient drugs for inhibiting mutant but not wild-type HSPCs. The zebrafish model described here could be exploited to identify small-molecule drugs already in human use that could be repurposed for the suppression of *ASXL1* mutant HSPCs and their progeny.

## MATERIALS AND METHODS

### Zebrafish maintenance

Wild-type stocks of AB fish, and transgenic and mutant lines were maintained according to a previously reported protocol (Bolli et al., 2010). Animal handling was approved by the Dana-Farber Institutional Animal Care and Use Committee. The *Tg*(*cmyb-EGFP)* line is described elsewhere (North et al., 2009), as are the details of a developmental staging system (Metscher and Ahlberg, 1999). For all experiments, zebrafish embryos were cultured in ‘egg water’ consisting of 0.03% sea salt and 0.002% Methylene Blue as a fungicide. To inhibit pigment formation and facilitate *in situ* hybridization, we incubated embryos with 0.0045% 1-phenyl-2-thiourea (Sigma).

### TALEN and targeting-vector construction and microinjection

The pair of TALENs recognizing exon 2 of the zebrafish *asxl1* gene was designed with ZiFiT Targeter software (http://zifit.partners.org/ZiFiT/), and the TAL effector repeats were constructed by the ‘unit assembly’ method described previously (Sander et al., 2011). TALEN mRNA was synthesized by *in vitro* transcription using the SP6 mMESSAGE mMACHINE Kit (Ambion). Approximately 50 pg of mRNAs encoding each of the two TALENs were injected into 1-cell-stage zebrafish embryos. The genomic DNA was isolated from a mixture of 3 embryos at 48 hpf, and the fragments containing the TALEN target site were amplified by PCR and sequenced with specific primers to examine the efficiency of the TALENs.

### Morpholinos and mRNA microinjection

*P53* morpholino, *bid* morpholino and control morpholinos were purchased from Gene Tools LLC (all the sequences are listed in Table S1). Capped mRNAs (*bcl2*-mCherry) were transcribed from linearized PCS2+ plasmids (mMessage Machine; Ambion), purified, and diluted to 100 ng/ml for injection at the 1-cell stage of development.

### Whole-mount *in situ* hybridization

Digoxigenin-labeled RNA probes were transcribed using linearized constructs with T3 or T7 polymerase (Ambion). Embryos at the desired time points were fixed overnight in 4% paraformaldehyde (PFA) at 4°C. Whole-mount *in situ* hybridization (WISH) was performed as described (North et al., 2009).

### Cell suspension preparation and flow cytometry

Adult fish were anesthetized with 0.02% tricaine before kidney removal. The kidney was dissected and placed in ice-cold 0.9×PBS containing 5% fetal calf serum (FCS). Single-cell suspensions were generated by aspiration, followed by mild teasing of the kidney on a 40-μm nylon mesh filter with a pipette tip. Blood cell populations were analyzed on a BD FACS Aria with high forward scatter/side scatter (FSC/SSC), as previously described (Traver et al., 2003). Data analyses were performed with FlowJo software (TreeStar, Ashland, OR, USA). Statistical analysis was done with Prism software with unpaired Student's *t*-tests to determine the *P*-value for each genotype group compared with controls.

### Peripheral blood cell counts

Adult fish were anesthetized with 0.02% tricaine, and 1 µl peripheral blood was obtained by cardiac puncture diluted in 499 µl of 0.9×PBS containing 5% FCS. Ten microliters of heparin sodium (1000 units/ml) was added to prevent blood coagulation, and 20 µl of Flow-Check fluorospheres (10^6^ fluorospheres/ml; Beckman Coulter, Inc.) was added to this solution. The number of cells corresponding to 1000 counted fluorospheres was obtained with use of the BDFACSAria with high FSC/SSC, as previously described (Gjini et al., 2015). Data analyses were performed with FlowJo software (TreeStar).

### Quantitative-PCR analysis

RNA from the clipped caudal hematopoietic tissue region from *asxl1^+/+^*, *asxl1^+/^^−^* and *asxl1^−/−^* embryos was extracted with TRIZOL reagent (Invitrogen). cDNA was synthesized with Superscript III (Invitrogen), while quantitative real-time PCR (qPCR) was carried out on a ViiA 7 real-time PCR system using SYBR Green. The fold change was calculated by the ΔΔCT method normalized to β-actin (see Table S1 for the primer sequences).

### Kidney smears and MGG staining

The kidney was dissected and smeared on glass slides, which were then fixed and stained with MGG stain (Sigma-Aldrich) according to the manufacturer's instructions and visualized with an Olympus BX51 microscope (Olympus).

### Phosphorylated histone H3 labeling and TUNEL assay

TUNEL was performed with the *In-Situ* Cell Death Detection kit (POD: Roche) according to the manufacturer's recommendations. Phosphorylated histone H3 labeling of fixed embryos was performed with the rabbit anti-phosphohistone H3 antibody (Santa Cruz) at 4°C overnight and visualized with Alexa-Fluor-488 goat anti-rabbit secondary antibody (Invitrogen).

Images of zebrafish immunofluorescence staining or live transgenic embryos were taken with a Leica SP5X scanning confocal microscope. The embryos were mounted in 1% low-melt agarose and the confocal images were captured with 20× objective. Fluorescence-positive cells were counted in each individual slice and sum numbers were analyzed with the GraphPad Prism 7 software using the two-tailed Student's *t*-test. The optical slice thickness is 3 µm.

### Genotyping

For embryo stages and adult stages, *asxl1* mutant fish were individually genotyped using one forward (5′-GGTGAATGTCTTTGCCGTTC-3′) and one reverse (5′-GAGAGTGAAGCATGGTGACAAG-3′) primer. The zebrafish *bim* mutant line (insertion mutation) was crossed with the *asxl1* mutant line in the rescue experiment. Genotyping primers are listed in Table S1.

## Supplementary Material

Supplementary information
