# Supplementary material for: Disruption of asxl1 results in myeloproliferative neoplasms in zebrafish
Source: Dis Model Mech. 2019 May 7;12(5):dmm035790. doi: 10.1242/dmm.035790 (PMC6550042; doi:10.1242/dmm.035790)
Supplement: Supplementary information [file dmm-12-035790-s1.pdf]

Fig. S1

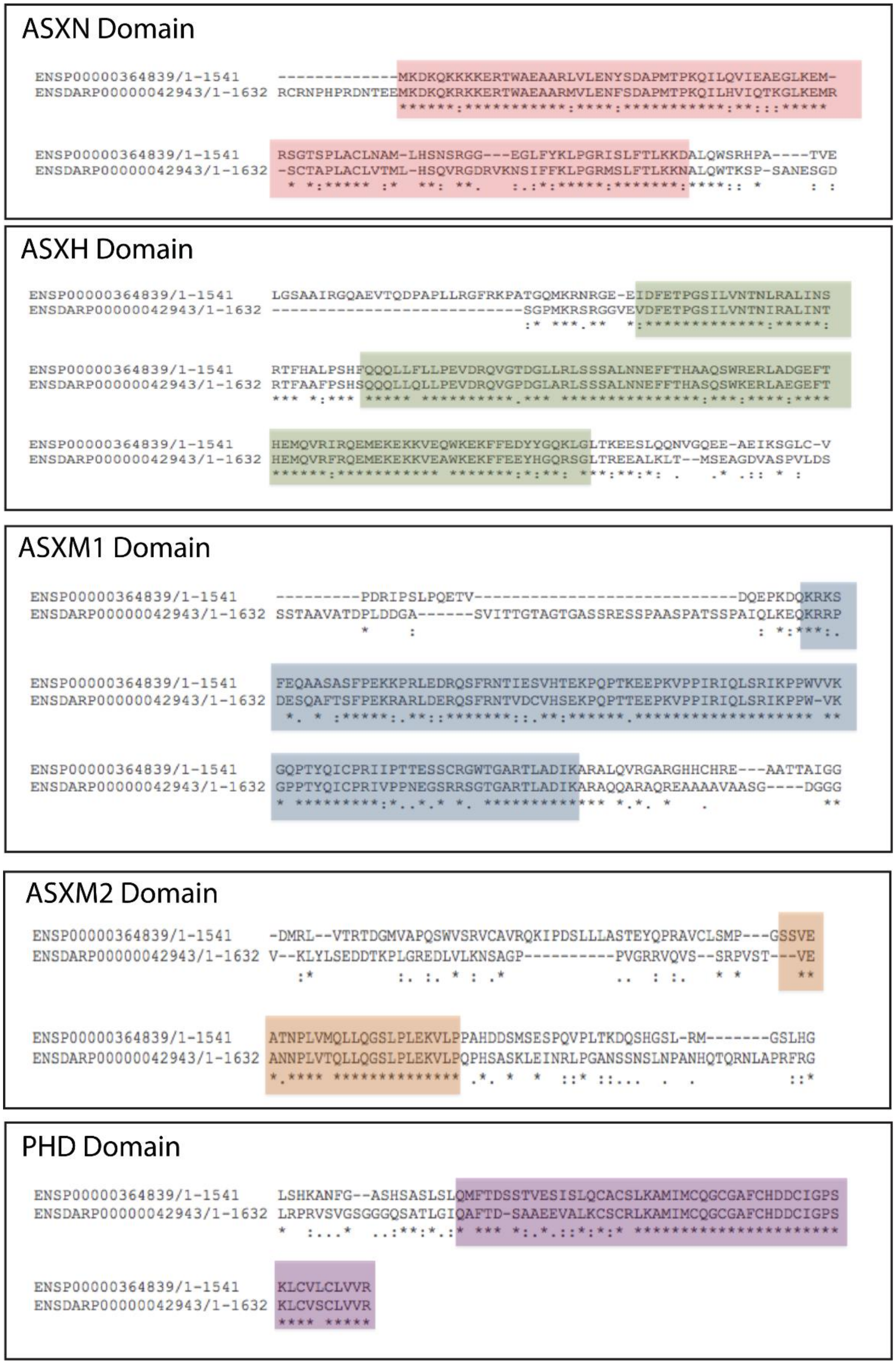

Fig. S1. Alignment of the predicted zebrafish and human proteins showed a high level of conservation for the ASXN, ASXH, ASXM1, ASXM2 and PHD domains.

Disease Models & Mechanisms • Supplementary information

Fig. S2

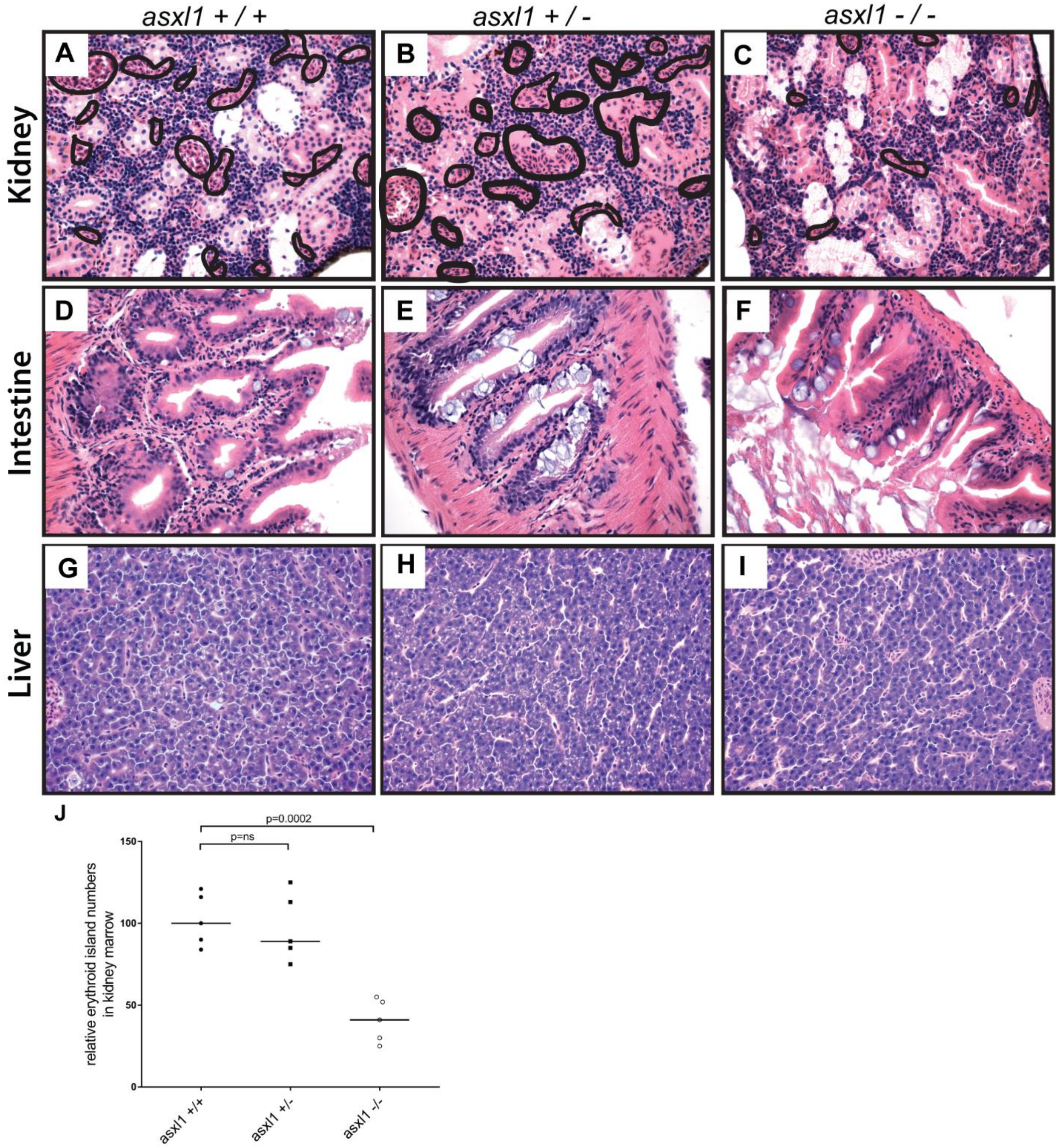

Fig. S2. Histopathological analysis of kidney, intestine and liver development in 17-month-old *asxl1+/+* *and asxl1+/-* and *asxl1-/-* fish. Results revealed that intestine (D, E and F) and liver (G, H and I) was normal and similar to that of the fish, indicating that these fish recovered from early developmental hypoplasia in these organs. However, *asxl1-/-* mutants did have decreased numbers of erythroid islands in the kidney marrow compared with *asxl1+/+ and asxl1+/-* fish (A, B and C), indicating that the hematopoietic system is abnormal in these animals. Area encircled with black line indicates erythroid islands. The quantification of erythroid islands was shown in (J), with the black bars representing the median values. ns p>0.05, *p<0.05, **p<0.01 by two-tailed unpaired t-test.

Disease Models & Mechanisms • Supplementary information

Fig.S3

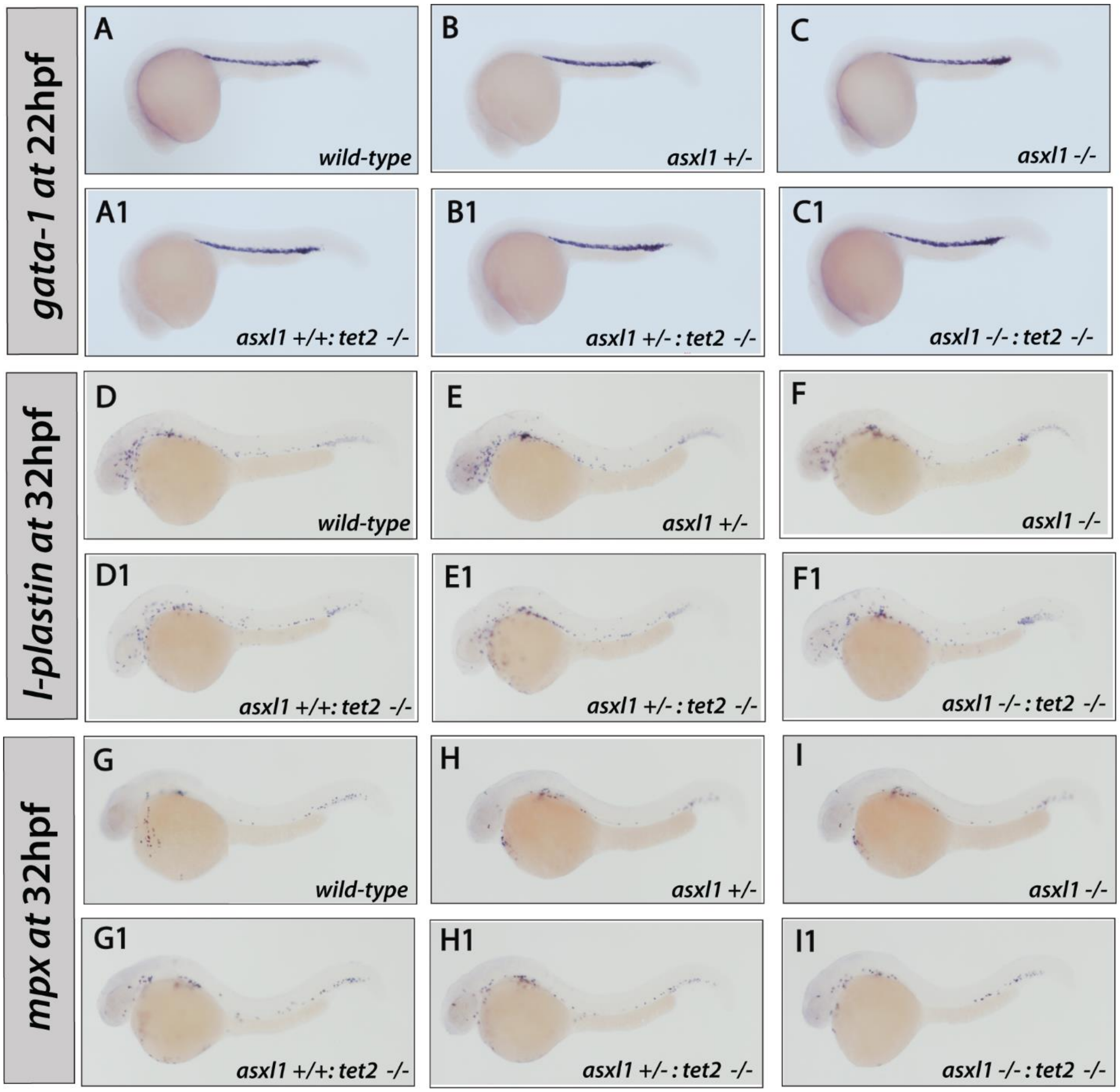

Fig. S3. Whole-mount *in situ* hybridization (WISH) for *gata-1* (22 hours-post-fertilization), *l-plastin and mpx* (22 hours-post-fertilization) was performed in *asxl1+/+*, *asxl1+/-*, *asxl1-/-*, *asxl1+/+tet2 -/-*, *asxl1+/-tet2 -/-* and *asxl1-/-tet2 -/-* zebrafish embryos. This assay shows that there is no difference in the number of cells expressing *gata-1, l-plastin and mpx* among all the different genotypes.

Disease Models & Mechanisms • Supplementary information

Fig.S4

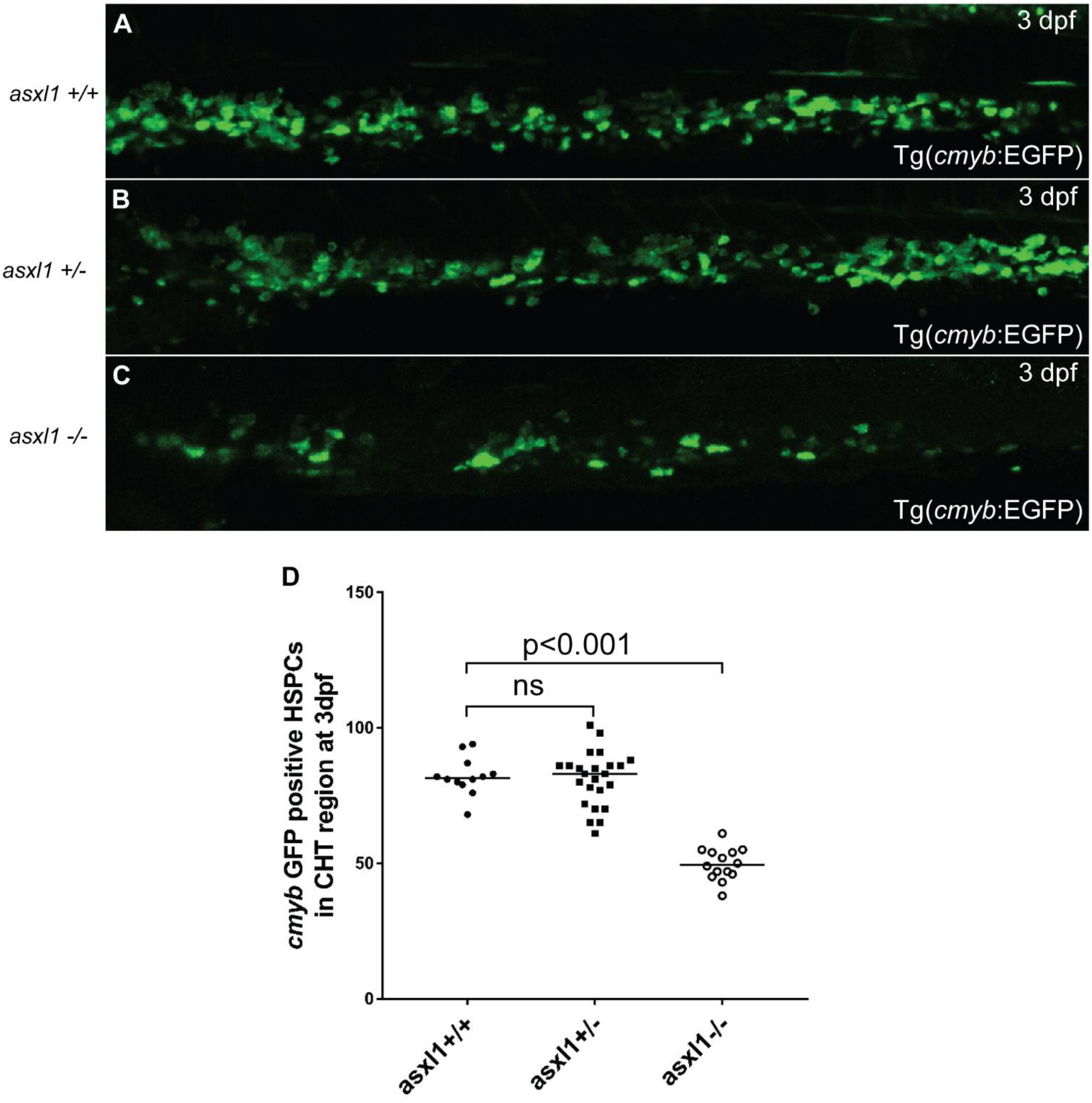

Fig. S4. The number of HSPCs in the CHT is reduced in the *asxl1* -/- embryos at 3 dpf. (A-C) EGFP+ HSPCs in *asxl1* +/-, *asxl1* +/- and *asxl1* -/- embryos. (D) Statistical analysis of HSPC numbers in *asxl1* +/-, *asxl1* +/- and *asxl1* -/- embryos indicate that EGFP+ HSPCs are decreased in CHT region in *asxl1* -/- embryos at 3 dpf.

Disease Models & Mechanisms • Supplementary information

Fig.S5

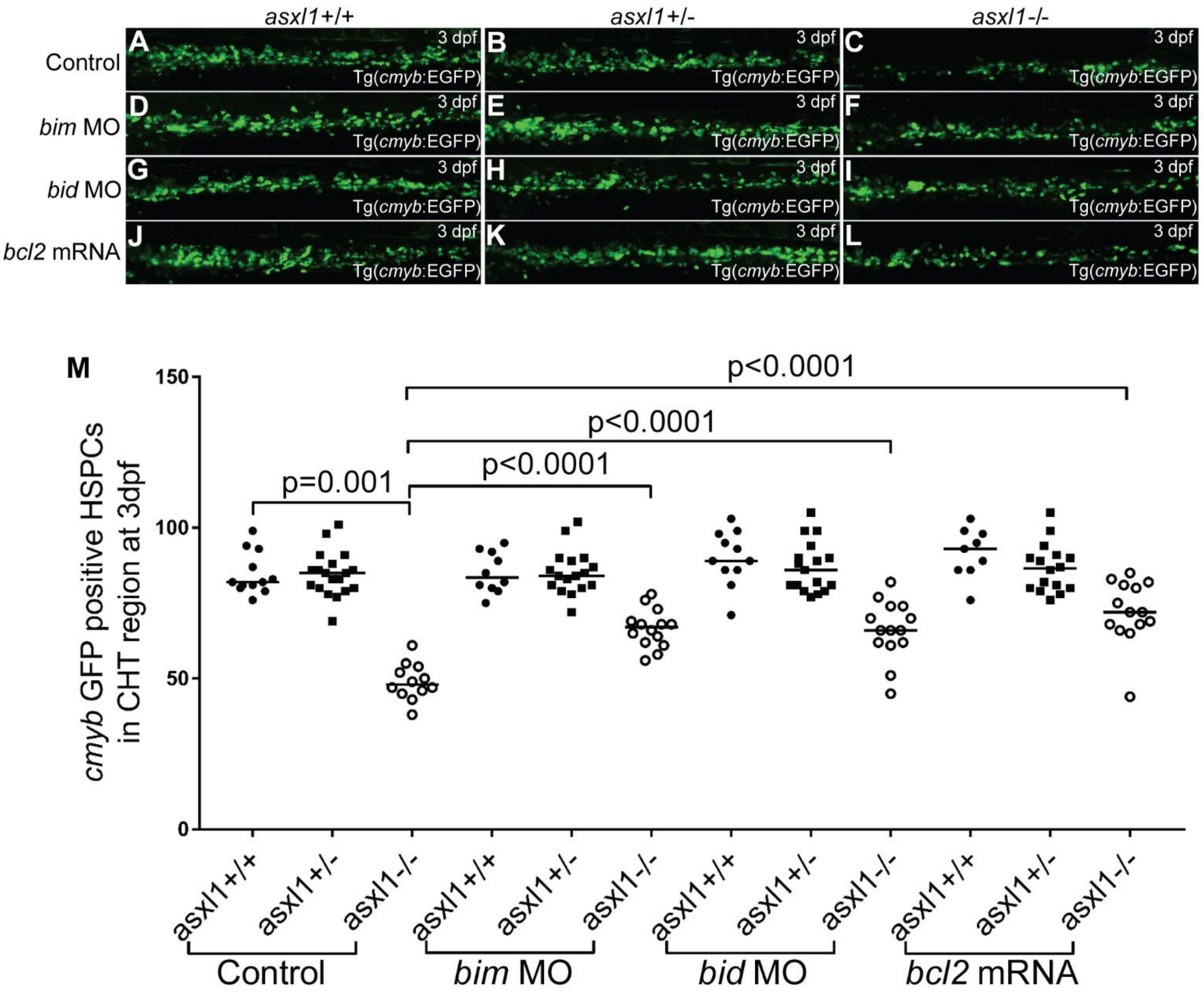

Fig. S5. EGFP+ HSPCs in *asxl1* +/+, *asxl1* +/- and *asxl1* -/- embryos at 3 dpf. Knockdown of *bim* (D-F) and *bid* (G-I) or overexpression of *bcl2* (J-L) each were able to partially rescue HSPC numbers in *asxl1* -/- embryos compared with control embryos. This result is expected because mitochondrial intrinsic programmed cell death depends on the combined activities of BH3-only pro-apoptotic proteins and is blocked by prosurvival proteins like bcl2.

Disease Models & Mechanisms • Supplementary information

Fig.S6

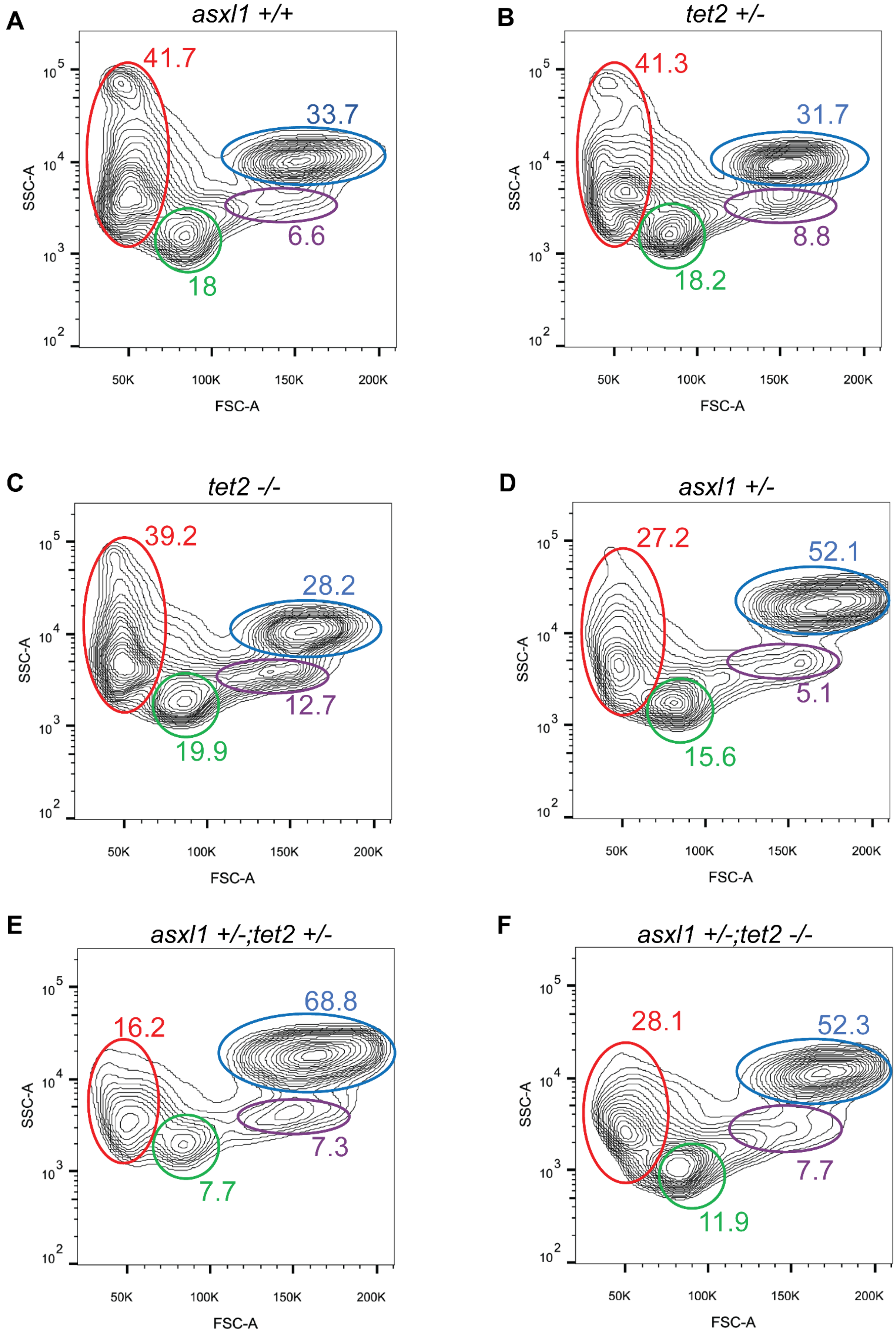

Disease Models & Mechanisms • Supplementary information

Fig. S6. Forward versus side scatter analysis plots for kidney marrow cell populations in 5-month-old. *asxl1* +/+ (A), *tet2* +/- (B), *tet2* -/- (C), *asxl1* +/- (D), *asxl1* +/-;*tet2* +/- (E) and *asxl1* +/-;*tet2* -/- (F). Erythrocyte cell contours are circled in red, lymphocyte cell contours are circled in green, myelomonocytes cell contours are circled in blue, and progenitor cell contours are circled in purple.

Table S1

Name	Sequence
p53 MO	GCGCCATTGCTTTGCAAGAATTG
Control MO	GGTCAGCATTCAAGAGACCATGCAT
bid MO	GGTCAAAGTTCCTGTTGAAGTCCAT
bim MO	TACTAAACTCCCGTTTGAACTCACC
bim genotyping-WT-f	GAGCAAACGCTGGCCAATGGCCCGG
bim genotyping-WT-r	GTCCGTCTTGCGCTTCGGAAATATT
bim genotyping-mutant-f	CGACAGCGATTCTGTGCCAGGTTC
bim genotyping-mutant-r	GACGCAGGCGCATAAAATCAGTC
p53 rt-f	ttcgagccactgccatctat
p53 rt-r	ttgccctccactcttatcaaa
puma rt-f	caggacagtctactcagggaca
puma rt-r	ctagcagatctggcagaggaa
bim rt-f	agttcaatcgcctctactgtga
bim rt-r	gacgatggcgtgttcgtt
bid rt-f	gcgacctacagagaccttttaca
bid rt-r	gagcctcttctgcattgactg
bax rt-f	gtttgcagcagatcggagat
bax rt-r	ggccactctgatgaagacg
bik rt-f	ctagaaagtgggcagcttgg
bik rt-r	tgccagggtgtatgtagtgc
bcl2 rt-f	tggcgtcccaggtagataat
bcl2 rt-r	accgtacatctccacgaagg
bcl Xl rt-f	cgtcctggcactacactgaa
bcl XI rt-r	tgtcatctgctttccacactg
mcl1 rt-f	gcaggactggatcctcaaaa
mcl1 rt-r	ccatgacggacaacagactc
b-actin rt-f	TACAATGAGCTCCGTGTTGC
b-actin rt-r	ACATACATGGCAGGGGTGTT

Table S1. All primers sequences used in this paper, including genotyping primers and real-time PCR primers.

Disease Models & Mechanisms • Supplementary information
